# Juxtapapillary Retinal Capillary Hemangioma: New Therapeutic Strategies

**Published:** 2014

**Authors:** Andrea Saitta, Michele Nicolai, Alfonso Giovannini, Cesare Mariotti

**Affiliations:** Department of Ophthalmology, Polytechnic University of Marche, Ancona, Italy

**Keywords:** Juxtapapillary Retinal Capillary Hemangioma, Therapeutic Strategies, Papillomacular Bundle

## Abstract

The treatment of juxtapapillary retinal capillary hemangiomas (JRCHs) is still a therapeutic dilemma without established guidelines. Because of the location of these hemangiomas on or adjacent to the optic nerve, treatment is difficult and complex, especially when JRCHs are located in the papillomacular bundle. This manuscript reviews the clinically relevant data on literature regarding the treatment of JRCHs, focusing on novel combined therapies that have shown promising results in these lesions.

## INTRODUCTION

Juxtapapillary retinal capillary hemangiomas (JRCHs) are vascular hamartomas that occur on or adjacent to the optic nerve head (1). Although JRCHs can occur sporadically as an isolated condition, most occur in association with von Hippel–Lindau (VHL) disease, an autosomal dominant neoplastic disorder (2). Three distinct growth types of JRCHs have been described, including the endophytic, exophytic, and sessile forms (3).

The clinical course of the JRCHs is usually progressive and difficult to predict. These tumors can start as small lesions at the optic disc or in the peripapillary area (most commonly on the temporal side of the disc) (1,2) If left untreated, JRCHs can grow and cause complications, such as exudation, subretinal fluid accumulation, macular edema, and exudative retinal detachment, resulting in visual deterioration (2-4). Furthermore, glial proliferation can lead to epiretinal membrane development or tractional retinal detachment. Rarely, these tumors regress spontaneously (5).

Because of the location of these hemangiomas on or adjacent to the optic nerve, treatment is very difficult. Although definitive treatment guidelines have yet to be established, in general, observation is chosen as the initial management of JRCHs in asymptomatic patients (6, 7). The treatment of these lesions should only be undertaken if vision is reduced or if there is lesion progression. The main goal of the treatment of JRCHs is to preserve visual acuity and the visual field without destruction of the function of the retina around the tumor. Treatment depends on the size, location and clinical manifestations of the hemangioma. In addition, juxtapapillary lesion therapies are often recalcitrant to treatment and the visual outcome after treatment varies. Several treatments have been proposed, such as laser photocoagulation, brachytherapy, transpupillary thermotherapy, photodynamic therapy (PDT), and surgical excision, but none of these treatments has proven to be particularly effective in inducing regression of the JRCHs (1-4, 6, 7).

Laser photocoagulation, brachytherapy and transpupillary thermoplasty have been shown to be effective in the treatment of optic disc hemangiomas, but all these approaches are risky and can result in permanent scotomas and poor clinical outcome due to the posterior location of the tumor and its proximity to the optic nerve (7-10).

More recently, PDT has been reported to be an alternative method to treat JRCHs because it enables a more selective vascular occlusion and appears to be less damaging to the optic disc.(11-14) Intravitreal anti-vascular endothelial growth factor (anti-VEGF) therapy alone or in combination with PDT has been used to treat JRCHs cases (15-19). Good results have been reported with these combined treatments (18, 19).

Vitreoretinal surgery can be a valid option when JRCHs are associated with epiretinal membrane formation and serous or tractional detachment. Further improvement of the treatment results of vitreoretinal surgery may be achieved by combination with PDT and with/without intravitreal anti-VEGF (20, 21).

This article reviews the current literature on safety and efficacy of several options for treatment of JRCHs, focusing on new combined treatments.

## Anti-angiogenic (anti-VEGF) agents for JRCHs

Several anti-VEGF agents (Pegaptanib, Bevacizumab, and Ranibizumab) have been used extensively for treatment of JRCH and have a relatively good safety profile but mixed treatment outcomes, suggesting that the general efficacy of anti-angiogenic agents in JRCHs is uncertain.(15-17) The rationale of anti-VEGF treatment is to achieve disease control by decreasing the growth and exudation of JRCHs through limiting the contribution of VEGF to these processes (22).

Intravitreal Ranibizumab, as monotherapy every 4 weeks, has shown beneficial effects with the smallest lesion with less exudation (16). Smaller lesions may have a higher rate of cell proliferation, and may be more sensitive to anti-angiogenic inhibition.

In addition, intravitreal anti-VEGF agents seem to have the advantage of a decreased potential for retinal damage compared with other treatments for JRCH (17). Anti-VEGF agents might therefore be considered as an alternative treatment for progressive JRCH, especially in patients with well-preserved visual acuity and visual field.

However, the overall results also indicate limitations of intravitreal anti-VEGF treatment as monotherapy: first, an effective dose required for JRCHs may need to be higher than the dose required for the treatment of choroidal neovascularization; and second, the route of administration through the vitreous may limit the access to tumor cells in the interior of a large endophytic lesion (22). In conclusion, intravitreal anti-VEGF treatment as monotherapy can be an option in particular to treat smaller JRCH without visual acuity loss. However, future prospective studies with longer follow-up and greater numbers of cases are needed to confirm the effectiveness of intravitreal anti-VEGF for JRCHs.

Also, many cases have been reported in which JRCHs were treated with systemic administration of the anti-VEGF (SU5416 and Bevacizumab), with transient reduction in exudation but with limited benefit (23, 24). Moreover, the systemic treatment could be associated with severe side effects that preclude its further use.

In summary, Wong et al. suggested the following future directions for anti-angiogenic therapy: the use of systemic anti-VEGF therapy, possibly combined with intravitreal therapy, to increase drug access to a large vascularized lesion; the targeting of multiple angiogenic molecules as suggested by the molecular biology of the disease (e.g. other angiogenic molecules upregulated with the loss of VHL function, such as platelet-derived growth factor (PDGF)); and the delivery of higher and more sustained levels of drug as may be affected by sustained release drug devices and intraocular gene therapy techniques (22).

**Figure 1:**
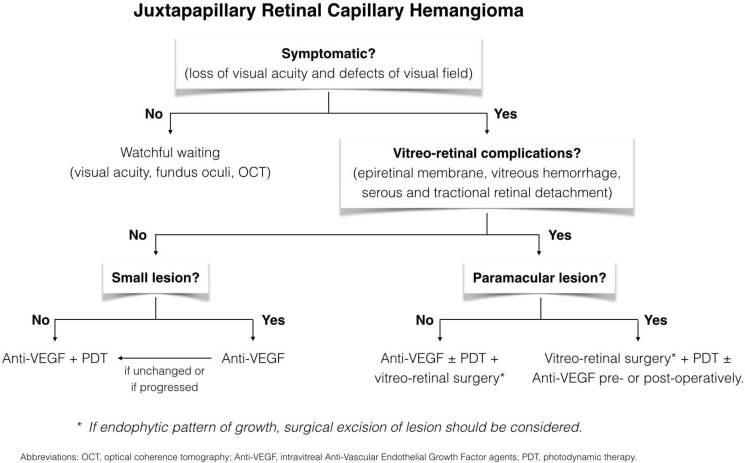
Algorithm approach of juxtapapillary retinal capillary hemangiomas

## Verteporfin photodynamic treatment (PDT) for JRCHs

In recent years, verteporfin PDT has been reported to be an alternative method to induce tumor regression or stabilization because it enables a more selective vascular occlusion and appears to be less damaging to the optic disc than laser photocoagulation (11-14). In particular, PDT was shown to be effective in causing fibrosis and involution of the smaller JRCH. For larger tumors, verteporfin may only be activated on the surface of the tumor, and the reactive oxygen species may not cause closure of deeper tumor vessels (12).

Photodynamic treatment for JRCH can be effective in treating macular edema and subretinal fluid, however, this may not translate into improvement in visual acuity in all cases. Also, the overall results indicate limitations of PDT as monotherapy. One possible major limitation of PDT is increased fibrosis with associated progression of epiretinal membrane (ERM) . In their series, Aaberg et al. theorized that while retinal traction may be an inevitable effect of tumor fibrosis following PDT, it might be modulated by inducing slower tumor regression using smaller repeated treatments (25). Other authors suggested different protocol parameters from standard to minimize the damage to the neural tissue (14, 21, 26). Other complications of PDT are transient optic disc edema, retinal vessel occlusion, optic neuropathy, vitreous hemorrhage, massive retinal detachment and massive subretinal hemorrhage (12, 25-27).

A larger prospective study is necessary to validate the efficacy and safety of PDT for JRCHs, particularly concerning the risk of ERM development and other complications. Important questions regarding the PDT dose modulation, treatment frequency, and number of treatments also remain unanswered. In addition, the exact reason for sensitivity of the tumor to treatment remains to be elucidated.

By combining anti-VEGF with reduced fluence PDT, the outline of the primary angioma can be better delineated and may thus reduce the energy and the treatment area, thereby minimizing the damage to the neurological tissues (18). Recent reports of combined therapy with anti-VEGF therapy and PDT have shown promising results in these lesions (18,19).

## Vitreoretinal surgery for JRCHs

Vitreoretinal surgery is still an option to consider for the treatment of progressive cases of JRCHs complicated with ERM formation, serous or tractional retinal detachment of the macula and vitreous hemorrhage from large tumors, which leads to a poor visual prognosis (6). Surgical excision can be a possible option only when tumor has an endophytic growth (28).

However, the visual recovery following surgery will only be maintained if the underlying tumors can be eradicated or their growth be interrupted.

In 2011, Gaudric et al. suggested that 20-gauge vitreoretinal surgery may be preceded or followed by conventional laser photocoagulation or PDT (29).

More recently, the minimally invasive sutureless transconjunctival pars plana vitrectomy (especially 25-gauge) has progressed so much that it has become the approach of choice for most vitreoretinal pathologies (30). Mariotti et al. reported a case of a progressive paramacular JRCH, with a sessile exophytic growth and associated with tractional macular detachment, that was managed successfully with 25-gauge vitreoretinal surgery, followed by two sessions of half-fluence PDT (21). After combined treatment, the patient had marked regression of the hemangioma, an increase in visual acuity, reduction of papillomacular area fluid, and macular drying that persisted at the 2 years follow-up visit. Some authors suggested the role of vitreoretinal surgery as first approach to improve safety and efficacy of PDT. Firstly, displacing tumor as more as possible from the head of the optic disc may minimize the risk of optic neuropathy after PDT. And secondly, removing glial proliferation over JRCH could improve penetration of Vysudine in inner part of tumor. However, more studies are necessary to validate these theories.

Fong et al. reported a case of an inferotemporal JRCH associated with tractional detachment of the macula that underwent successful combined therapy with intravitreal ranibizumab injection and PDT one week before vitreoretinal surgery (20).

In similar cases, combining therapy of PDT and intravitreal anti-VEGF one week before surgery may be useful in reducing the macular edema and the risk of retinal cyst rupture during peeling procedure. In addition, pre-operative anti-VEGF agents may reduce tumor vascularization and intraoperative bleeding (20).

## CONCLUSION

The treatment of JRCHs remains controversial. Because of the location of these hemangiomas on or adjacent to the optic nerve, treatment is difficult and complex. In addition, the complexity of performing a prospective study in JRCHs has been a major barrier to date.

Several small case series have been reported that demonstrate the inconstant clinical course and treatment outcomes of these patients. In general, data presented in these studies suggest that monotherapies with anti-angiogenic agents or with PDT are inadequate to treat JRCHs, especially for larger and/or complicated lesions. In addition, the exact reason for responsiveness of the tumor to several treatments remains to be elucidated.

More recently, case reports with combined therapies (PDT plus anti-VEGF; vitreoretinal surgery plus PDT plus anti-VEGF) have shown promising results in these lesions. A more systematic investigation would support the perceived importance of these combined therapies in the management of JRCHs.

According to data in literature, we propose a decision tree summarizing recommended approach to the treatment of JRCHs. (Figure 1) However; future prospective studies are needed to verify these hypotheses and to determine successful therapeutic strategies for JRCHs.

New insights into the underlying mechanisms of tumor formation and greater knowledge of the natural history of the eye disease in VHL disease should lead to improved future treatments. In addition, an improved genetic and molecular understanding of how VHL dysfunction results in ocular disease and the development of an animal model for ocular VHL disease will be the next substantive steps forward in the generation of new therapies for JRCHs (22).
